# Xanthine dehydrogenase as a prognostic biomarker related to tumor immunology in hepatocellular carcinoma

**DOI:** 10.1186/s12935-021-02173-7

**Published:** 2021-09-08

**Authors:** Zhen Lin, Yi-Zhao Xie, Ming-Chun Zhao, Pin-Pin Hou, Juan Tang, Guang-Liang Chen

**Affiliations:** 1 Department of Oncology, First Affiliated Hospital, College of Medicine, Zhejiang University, Hangzhou, 310003 China; 2 Department of Internal Medicine 3, Friedrich-Alexander-University Erlangen-Nürnberg (FAU) and Universitätsklinikum Erlangen, 91054 Erlangen, Germany; 3 Department of Medical Oncology, Fudan University, Shanghai Cancer Center, Shanghai, 200032 China; 4 Department of Oncology, Shanghai Medical College of Fudan University, Shanghai, 200032 China; 5 Department of Pathology, Guilin Hospital of Chinese Traditional and Western Medicine, Guilin, 541004 China; 6 Central Laboratory, Renji Hospital, Shanghai Jiao Tong University School of Medicine, Shanghai, 201114 China; 7 Department of Pathology, The Second Affiliated Hospital of Guilin Medical University, Guilin, 541199 China

**Keywords:** XDH, Hepatocellular carcinoma, Biomarker, Immunity, Exhaustion marker

## Abstract

**Background:**

Xanthine dehydrogenase (XDH) is a critical enzyme involved in the oxidative metabolism of purines, pterin and aldehydes and a central component of the innate immune system. However, the prognostic value of XDH in predicting tumor-infiltrating lymphocyte abundance, the immune response, and survival in different cancers, including hepatocellular carcinoma (HCC), is still unclear.

**Methods:**

XDH expression was analyzed in multiple databases, including Oncomine, the Tumor Immune Estimation Resource (TIMER), the Kaplan–Meier plotter database, the Gene Expression Profiling Interactive Analysis (GEPIA) database, and The Cancer Genome Atlas (TCGA). XDH-associated transcriptional profiles were detected with an mRNA array, and the levels of infiltrating immune cells were validated by immunohistochemistry (IHC) of HCC tissues. A predictive signature containing multiple XDH-associated immune genes was established using the Cox regression model.

**Results:**

Decreased *XDH* mRNA expression was detected in human cancers originating from the liver, bladder, breast, colon, bile duct, kidney, and hematolymphoid system. The prognostic potential of XDH mRNA expression was also significant in certain other cancers, including HCC, breast cancer, kidney or bladder carcinoma, gastric cancer, mesothelioma, lung cancer, and ovarian cancer. In HCC, a low *XDH* mRNA level predicted poorer overall survival, disease-specific survival, disease-free survival, and progression-free survival. The prognostic value of XDH was independent of the clinical features of HCC patients. Indeed, XDH expression in HCC activated several immune-related pathways, including the T cell receptor, PI3K-AKT, and MAPK signaling pathways, which induced a cytotoxic immune response. Importantly, the microenvironment of XDH^high^ HCC tumors contained abundant infiltrating CD8 + T cells but not exhausted T cells. A risk prediction signature based on multiple XDH-associated immune genes was revealed as an independent predictor in the TCGA liver cancer cohort.

**Conclusion:**

These findings suggest that XDH is a valuable prognostic biomarker in HCC and other cancers and indicate that it may function in tumor immunology. Loss of XDH expression may be an immune evasion mechanism for HCC.

**Supplementary Information:**

The online version contains supplementary material available at 10.1186/s12935-021-02173-7.

## Background

Hepatocellular carcinoma (HCC) is the seventh most common tumor and the third most common cause of cancer-related death worldwide [1]. Patients with HCC are often diagnosed at advanced stages or are not eligible for curative treatments [2, 3]. Despite optimal treatment, the high recurrence rate of HCC remains a significant concern [4]. The overall prognosis of HCC is poor, with a 5-year overall survival estimated at 10–18% [5].

The major risk factors for developing HCC include chronic hepatitis B or C virus infections, nonalcoholic steatohepatitis, and alcohol-related liver diseases [6]. Indeed, HCC is strongly influenced by the immune system [7]. The background of chronic inflamed livers leads to tumor development and is associated with an immune-rich contexture in the HCC microenvironment [8–10]. Recently, developed immune-based therapies for patients with advanced HCC represent a promising treatment option [5, 9]. However, the complex interface between inflammation, fibrosis, and the immune response involved in HCC pathogenesis is poorly understood [11].

Xanthine dehydrogenase (XDH) functions as a key regulator of purine metabolism [12, 13], inflammatory cascades [14], and the innate immune system [15]. In fact, XDH is widely expressed in human tissues, with high levels in the liver [16–20]. Decreased XDH activity is believed to contribute to the development and progression of HCC [21, 22]. Indeed, it has been reported that decreases in XDH activity levels are associated with poor prognoses for cancers, including breast cancer [23], gastric cancer [24], ovarian cancer [25], non-small-cell lung cancer [26] and colorectal cancer [27]. Despite these intriguing findings, how decreased activity or expression of XDH regulates tumor immunobiology in the development and progression of cancers, including HCC, remains poorly understood.

In this study, we found that the expression of XDH has significant prognostic implications in different types of tumors, including HCC. Moreover, the XDH-associated signaling pathway may regulate the immune response and tumor-infiltrating immune cells in HCC. Importantly, the prognostic value of the XDH-associated immune signature in HCC shed light on XDH might modulate tumor immunity in HCC.

## Methods and materials

### Data acquisition

The Oncomine database was used to determine the expression level of the *XDH* gene in various types of cancers (https://www.oncomine.org/resource/login.html) [28]. The screening criteria were as follows: P-value of 0.001, fold change of 1.5, and gene ranking of all. Kaplan–Meier plotter was used to assess the effect of 54,675 genes on survival using 10,461 cancer samples. The correlation between XDH expression and survival in breast, ovarian, lung and gastric cancers was analyzed by Kaplan–Meier plotter (http://kmplot.com/analysis/) [29]. Hazard ratios (HRs) with 95% confidence intervals and log-rank P-values were also computed. The Tumor IMmune Estimation Resource (TIMER) is a comprehensive resource for the systematic analysis of immune infiltrates across diverse cancer types (https://cistrome.shinyapps.io/timer/) [30]. TIMER applies a previously published statistical deconvolution method [31] to infer the abundance of tumor-infiltrating immune cells from gene expression profiles. The TIMER database assesses 10,897 samples from The Cancer Genome Atlas (TCGA) to estimate the abundance of immune infiltrates, including B cells, CD4 + T cells, CD8 + T cells, neutrophils, macrophages, and dendritic cells (DCs) [32]. In addition, correlations between the expression levels of XDH and those of gene markers of tumor-infiltrating immune cells were explored via correlation modules. Gene Expression Profiling Interactive Analysis (GEPIA) (http://gepia.cancer-pku.cn/index.html) is an interactive website that includes data for 9736 tumors and 8587 normal samples from the TCGA and Genotype-Tissue Expression (GTEx) projects and analyzes RNA sequencing expression [33]. GEPIA was used to generate survival curves, including overall survival (OS) and disease-free survival (DFS) curves, based on gene expression with the log-rank test and the Mantel-Cox test in 25 different types of cancer. Gene expression correlation analysis was performed to obtain sets of TCGA expression data. The Spearman method was used to determine the correlation coefficient.

### RNA microarray

As in our previous report [21], MHCC-97H cells were transfected with plasmids for overexpressing XDH or a pEZ-Lv201 control vector (Genecopoeia, Guangzhou, China), which served as a negative control. RNA was extracted from the tumors using the Qiagen RNeasy Midi Kit according to the manufacturers' instructions. Complementary RNA (cRNA) was prepared according to the GeneChip Expression Analysis Technical Manual (Affymetrix), hybridized onto Affymetrix Human U133 Plus 2.0, and scanned by a GeneChip Scanner 3000 (Affymetrix). Differential gene expression analysis between treatment groups was calculated using the R limma package [34]. For functional annotation, we used pathways from the Kyoto Encyclopedia of Genes and Genomes (KEGG) and gene ontology (GO) databases as provided by the clusterprofiler package [35]. P-values were corrected for multiple testing using the Benjamini–Hochberg method. The GSEA algorithm [36], which tests whether a gene set is significantly perturbed relative to all genes, was applied for analysis.

### Western blotting

The preparation of cell lysates and the western blotting procedure were carried out as previously indicated [21, 37]. Briefly, whole-cell lysates were separated by 8–10% sodium dodecyl sulfate–polyacrylamide gel electrophoresis. Equal amounts of resolved proteins were transferred to polyvinylidene difluoride (PVDF) membranes. After incubation with QuickBlock blocking buffer (Beyotime Biotechnology, China, cat. no. P0233), the membranes were then probed with specific primary antibodies (Additional file 1: Table S1) and secondary antibodies (Beyotime Biotechnology, China). Images of the bands were acquired with an Amersham Imager 600 (GE Healthcare, Russellville, AR, USA).

### Immunohistochemical analysis

The immunohistochemical staining procedure was conducted as previously described [21, 37, 38]. All patients provided their written informed consent. HCC liver samples were obtained according to a protocol approved by the ethics committee of Fudan University. Immunoreaction images were viewed and captured by Motic DSAssistant software (Motic VM V1 Viewer 2.0). To quantify the density of tumor-infiltrating immune cells, the three most representative areas of stroma were evaluated at 200 × magnification, and the mean value was adopted [39]. For immune cell markers (CD3, CD4, CD8, CD20, CD68 and PD-1) (Additional file 1: Table S2), the counts of all positive cells by immunostaining were recorded in terms of cells/mm^2^. Two pathologists who were blinded to the patient outcomes independently evaluated the immunohistochemistry (IHC) staining of each sample.

### Construction of prognostic signature

To develop an XDH-associated prognostic signature including multiple immune genes, a comprehensive list of immune-related genes was downloaded from the Immunology Database and Analysis Portal (ImmPort) database (https://immport.niaid.nih.gov). The Pearson coefficient between *XDH* and these immune genes was calculated, and an absolute value of coefficient > 0.3 with P < 0.05 was set as the identification criterion. Then, stepwise variable selection was performed with the Akaike information criterion in the Cox model [40]. After the immune genes were chosen, the prognostic index, referred to as the risk score, was calculated as follows: risk score = β_1_x_1_ + β_2_x_2_ + … + β_i_x_i_. In this formula, x_i_ is the expression level of each gene, while βi is the risk coefficient of each gene derived from the Cox model [41]. Multivariate analysis was performed for the risk score with adjustment for age, sex, T stage, N stage, M stage, and tumor-node-metastasis (TNM) stage. Time-dependent receiver operating characteristic (ROC) curves were adopted to determine the prognostic accuracy of the risk score using the timeROC package [42]. Then, a nomogram was built by incorporating the clinical characteristics and the risk scores of HCC patients, and the prognostic value of the nomogram was evaluated [43]. The nomogram was created via the rms package for R software. With the application of the bootstrap method (1000 replicates), a calibration curve was used to visualize the deviation of predicted probabilities from the actual values. The concordance index (C-index) was used to measure the predictive accuracy of the nomogram.

### Statistical analysis

Survival curves were generated by Kaplan–Meier plotter. The results generated in Oncomine are displayed with P-values, fold changes, and ranks. The results of Kaplan–Meier plots and GEPIA are displayed with HR and P or Cox P-values from a log-rank test. The gene expression correlations were evaluated by Spearman’s correlation test and statistical evaluation. P-values < 0.05 were considered to indicate statistical significance. Significant changes are represented as follows: *p < 0.05; **p < 0.01; ***p < 0.001. Nonsignificant changes are labeled as ns.

## Results

### Decreased expression of XDH mRNA in certain types of human cancer.

**Fig. 1:**
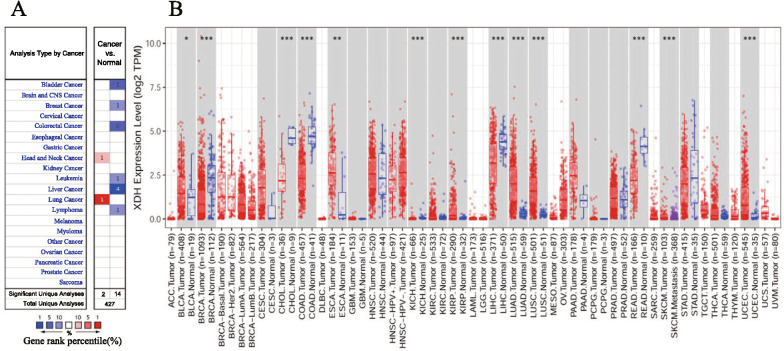
Xanthine dehydrogenase (XDH) expression levels in different types of human cancers. **A** A snapshot of XDH mRNA expression levels in 20 different types of cancers based on the Oncomine database. The datasets displayed in the colored rectangles are those with statistically significant XDH mRNA overexpression (right) and underexpression (left) in cancer tissue versus normal tissue. The threshold was set as follows: p-value of 0.001, fold change of 1.5, and gene ranking of all. **B** Human XDH expression levels in different tumor types from The Cancer Genome Atlas (TCGA) database were determined by the TIMER database (*p < 0.05, **p < 0.01, ***p < 0.001)

To profile *XDH* mRNA expression in tumor tissues and adjacent normal tissues across several types of cancer, we analyzed the Oncomine database and RNA-seq data of multiple cancers in the TCGA database. In the Oncomine database, lower *XDH* mRNA expression was detected in bladder cancer, breast cancer, colorectal cancer, leukemia, liver cancer, and lymphoma (Fig. 1A), while higher mRNA expression of *XDH* was observed in head and neck cancer (HNSC) and lung cancer in some datasets compared to that in normal tissues. The detailed results of XDH expression in different cancer types are summarized in Additional file 1: Table S3. Consistently, in TCGA, low *XDH* mRNA expression was significantly associated with breast invasive carcinoma (BRCA), colon adenocarcinoma (COAD), liver hepatocellular carcinoma (LIHC), and rectum adenocarcinoma (READ), while *XDH* mRNA expression was higher in HNSC, lung adenocarcinoma (LUAD), and lung squamous cell carcinoma (LUSC) tissues than in adjacent normal tissues (Fig. 1B). Additionally, in TCGA, *XDH* mRNA expression was lower in kidney chromophobe (KICH) and cholangiocarcinoma (CHOL) tumor tissues and higher in esophageal carcinoma (ESCA) and uterine corpus endometrial carcinoma (UCEC) than in adjacent normal tissues. However, *XDH* mRNA expression was higher in bladder urothelial carcinoma (BLCA). In summary, a decrease in the level of XDH mRNA was detected in human cancers originating from the liver, bladder, breast, colon, bile duct, kidney, and hematolymphoid system.

### XDH mRNA expression is associated with prognosis.

**Fig. 2:**
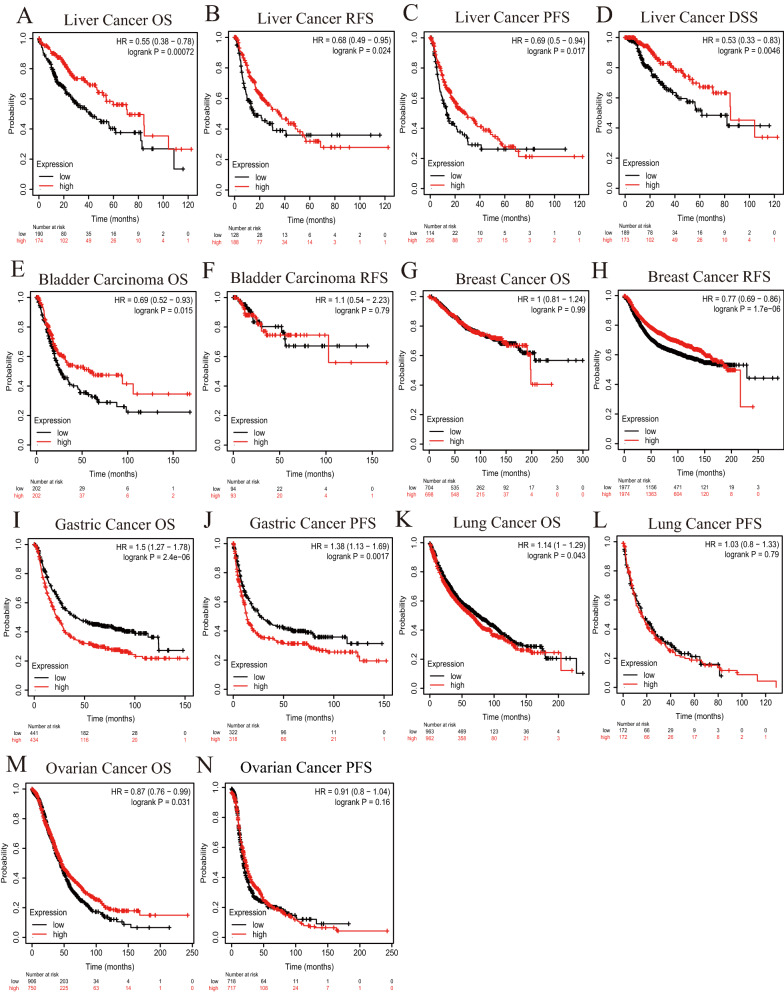
Kaplan–Meier analysis of survival according to XDH expression status in patients with different types of cancers using Kaplan–Meier plotter (A–N). **A**–**D** High XDH expression was correlated with worse overall survival (OS), progression-free survival (PFS), recurrence-free survival (RFS), and disease-specific survival (DSS) in hepatocellular carcinoma (HCC) cohorts (n = 364, n = 316, n = 370, n = 362). **E**–**F** Survival curves for OS and RFS in the bladder cancer cohort (n = 404, n = 187). **G**, **H** Survival curves for OS and RFS in the breast cancer cohort (n = 1402, n = 3951). **I**, **J** Survival curves for OS and RFS in the gastric cancer cohort (n = 875, n = 498). **K**, **L** Survival curves for OS and RFS in the lung cancer cohort (n = 1925, n = 344). **M**, **N** Survival curves for OS and RFS in the ovarian cancer cohort (n = 1656, n = 1435)

To investigate the impact of *XDH* mRNA expression on outcomes in patients with cancer, the correlations between *XDH* expression and OS prognosis and disease progression were evaluated using Kaplan–Meier plotter data based on Affymetrix microarrays. In HCC, patients with a high *XDH* mRNA expression level had significantly prolonged OS (hazard ratio (HR) 0.55, 95% confidence interval (CI) 0.38 to 0.78, P = 0.00072) and slower disease progression (recurrence-free survival (RFS) HR 0.68, 95% CI 0.49 to 0.95, P = 0.024; progression-free survival (PFS) HR 0.69, 95% CI 0.5 to 0.0.94, P = 0.017; disease-specific survival (DSS) HR 0.53, 95% CI 0.33 to 0.83, P = 0.0045) than patients with a low *XDH* mRNA expression level (Fig. 2A–D). In addition, high *XDH* mRNA expression was also correlated with better OS in bladder carcinoma (HR 0.69, 95% CI 0.52 to 0.93, P = 0.015) (Fig. 2E, F) and ovarian cancer (HR 0.87, 95% CI 0.76 to 0.99, P = 0.031) (Fig. 2M, N) and better RFS in breast cancers (HR 0.77, 95% CI 0.69 to 0.86, P = 1.7e−6, Fig. 2G, H). However, high *XDH* mRNA expression was associated with worse OS in patients with gastric cancer and lung cancer (Fig. 2I–L). Furthermore, the prognostic potential of *XDH* mRNA expression in 25 types of human cancers was assessed via the GEPIA website (Additional file 1: Figure S1). Indeed, high *XDH* mRNA expression was associated with a better prognosis in terms of OS in LIHC and BLCA. Moreover, high *XDH* mRNA expression was correlated with a worse prognosis in terms of OS and DFS in mesothelioma (MESO) and in terms of DFS in LUSC. Together, these findings suggest that the level of expression of *XDH* mRNA has a significant impact on the prognosis of specific human cancers.

### XDH mRNA expression correlates with the clinical characteristics of HCC patients.

**Fig. 3:**
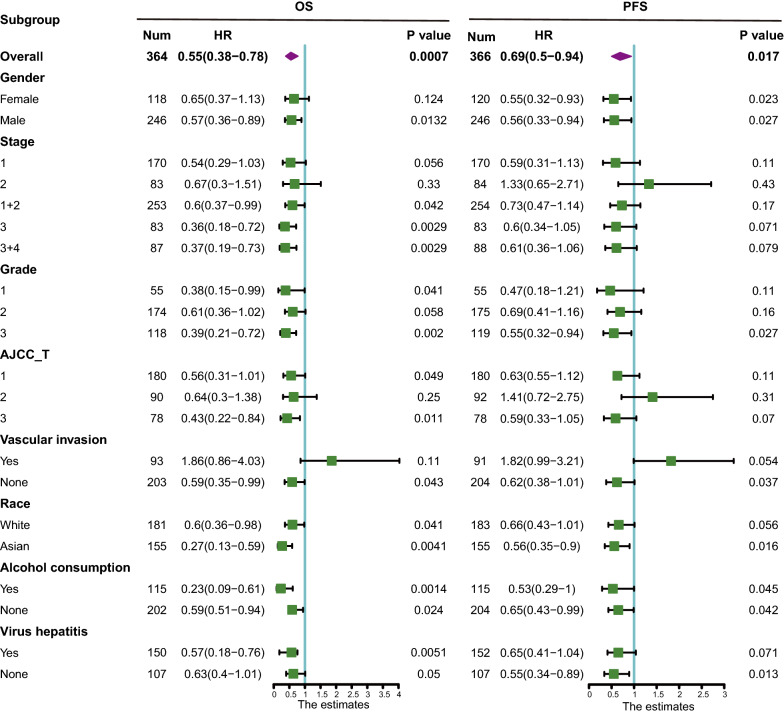
Analysis of the correlation between XDH mRNA expression and prognosis in HCC patients with different clinicopathological factors according to Kaplan–Meier plotter

We previously reported that decreased *XDH* mRNA expression is associated with aggressive HCC phenotypes [21]. In humans, XDH is mainly expressed in hepatic tissues [44]. Therefore, it is rational to consider liver cancers as a representative human cancer with weak XDH expression. To better understand the role of XDH expression in cancers, we investigated the relationship between XDH expression and the clinical characteristics of HCC patients in the Kaplan–Meier plotter database (Fig. 3). Indeed, low *XDH* mRNA expression correlated with poorer OS and PFS in males (OS: HR 0.57, P = 0.0132; PFS HR 0.56, P = 0.027), Asians (OS: HR 0.27, P = 0.0041; PFS HR 0.56, P = 0.016), alcohol consumers (OS: HR 0.23, P = 0.0014; PFS HR 0.53, P = 0.045), nonconsumers of alcohol (OS: HR 0.59, P = 0.024; PFS HR 0.65, P = 0.042), patients without vascular invasion (OS: HR 0.27, P = 0.0041; PFS HR 0.56, P = 0.016) and patients without hepatitis viral infection (OS: HR 0.63, P = 0.05; PFS HR 0.55, P = 0.013). Additionally, low *XDH* mRNA expression correlated with worse OS and PFS in HCC patients with grade 3 disease (OS: HR 0.39, P = 0.002; PFS: HR 0.55, P = 0.027). However, *XDH* mRNA expression was not significantly correlated with OS and PFS in stage 1 or stage 2 patients or patients with vascular invasion. Taken together, these results show the prognostic significance of XDH expression in HCC patients based on their clinical characteristics, particularly in those with advanced-stage HCC.

### XDH mRNA expression is related to tumor-infiltrating immune cells.

**Fig. 4:**
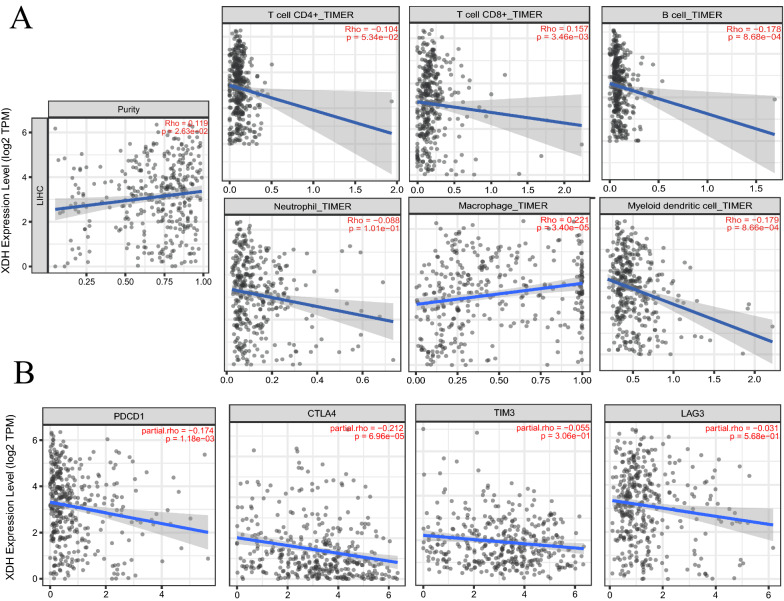
Analysis of the correlation between XDH expression and immune cell infiltration levels in HCC tissue using the Tumor IMmune Estimation Resource (TIMER) database. **A**. XDH expression had a significant positive correlation with tumor purity (rho = 0.119, p = 0.026) and CD8 + T cells (rho = 0.157, p = 0.003) and a significant negative correlation with the levels of infiltrating B cells (rho = − 0.178, p = 0.001) and DCs (rho = − 0.179, p = 0.001). **B** XDH was correlated with the exhausted T cell markers PDCD1 (r = 0.174, p = 1.18e−3) and CTLA4 (r = − 0.212, p = 6.96e−5)

Recently, several clinical trial results showed that immunotherapy is a promising therapy in HCC [5, 45]. In addition, the predictive value of tumor-infiltrating lymphocytes in lymph node metastases and survival has been validated in cancers, including HCC [46, 47], gastric cancer [48], and cutaneous melanoma [49]. Next, we described the relationship between the expression of XDH and infiltrating immune cells in 40 cancer types, including HCC, using the TIMER database. The results demonstrated that XDH expression was significantly correlated with tumor purity in 18 types of cancer. In addition, XDH expression was associated with infiltrating levels of CD4+ T cells in 9 cancer types, B cells in 12 cancer types, CD8+ T cells in 16 cancer types, DCs in 22 cancer types, macrophages in 9 cancer types, and neutrophils in 19 cancer types (Additional file 1: Table S4). For HCC, we observed that the level of XDH expression was positively correlated with the infiltration levels of CD8+ T cells (r = 0.157, p=3.46e−03) and macrophages (r = 0.221, p = 3.40e−05) but negatively correlated with the infiltration levels of B cells (r = − 0.178, p = 8.68e−04) and myeloid DCs (r = − 0.179, p=8.66e−04; Fig. 4A). As there is no estimate of exhausted T cells in the TIMER algorithm, we selected several marker genes representing tumor-infiltrating exhausted T cells [50]. The expression of XDH negatively correlated with that of programmed cell death protein 1 (PD1) (r = − 0.174, p = 1.18e−03) and cytotoxic T-lymphocyte-associated protein 4 (CTLA4) (r = − 0.212, p = 6.96e−05). Furthermore, a negative trend was also observed in T cell immunoglobulin and mucin domain-containing protein 3 (TIM3) and lymphocyte-activation gene 3 (LAG-3) (Fig. 4B). Together, these findings suggest that XDH may modulate the infiltration of immune cells into tumor tissues.

### XDH correlates with the immune response in HCC.

**Fig. 5:**
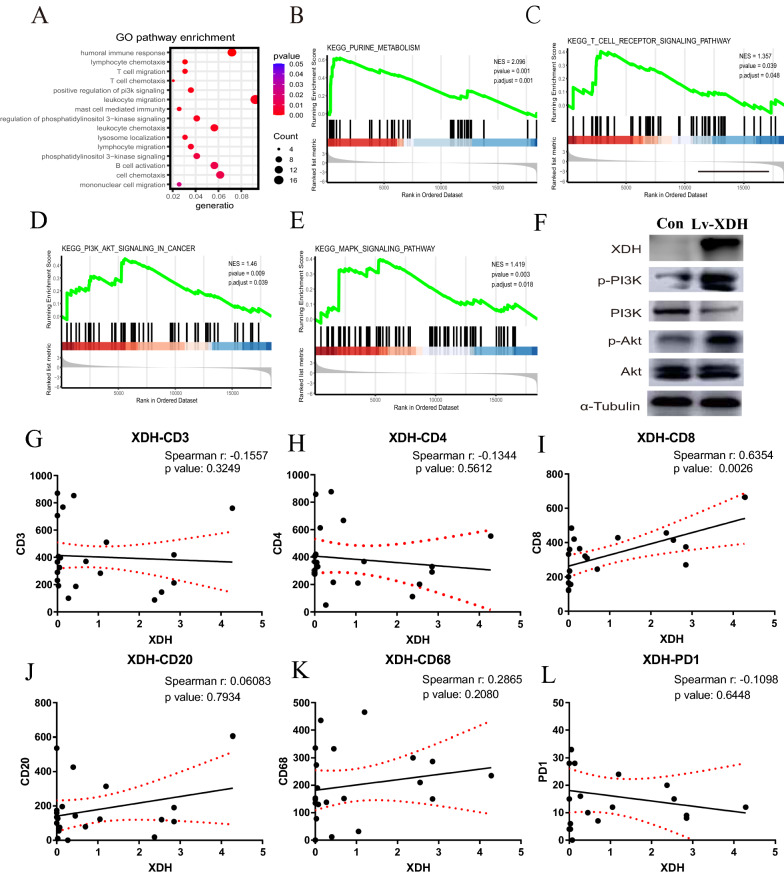
XDH expression was correlated with high immune cytotoxic activity. **A** Gene ontology (GO) enrichment analysis showing the significantly affected GO items. GO terms with P values less than 0.05 were considered significantly enriched. **B**–**E** Gene set enrichment analysis (GSEA) of the Kyoto Encyclopedia of Genes and Genomes (KEGG) database showed that the purine metabolism pathway, T cell receptor pathway, PI3K-Akt pathway and MAPK pathway were activated. F Western blot analysis of the expression of XDH and PI3K-AKT pathway proteins. G–L IHC showed the correlation between the protein levels of XDH and those of other immune cell markers

To understand the mechanistic role of XDH, we performed in-depth analysis of the relationship between XDH expression and tumor-infiltrating immune cells. To profile XDH-related gene expression, XDH was overexpressed in an HCC cell line (MHCC-97H), and the cells were subjected to RNA array analysis. Differential gene expression analysis (overexpression group versus control group) was performed with the limma package. GO enrichment analysis showed that the immune response was upregulated by XDH (Fig. 5A). GSEA confirmed that XDH overexpression upregulated the purine metabolism pathway and activated several immune-related pathways, including the T cell receptor, PI3K-AKT, and MAPK signaling pathways (Fig. 5B, E). Indeed, western blot analysis confirmed that XDH overexpression induced the activation of the PI3K-Akt pathway (Fig. 5F; Additional file 1: Figure S2; Additional file 3). To clarify the correlation between XDH expression and immune cell markers in HCC tumor tissue, IHC analysis of 6 types of immune cells was performed (Fig. 5; Additional file 1: Figure S3; Additional file 2). As shown in Fig. 5I, the expression of XDH was positively correlated with the infiltration of CD8 + immune cells (r = 0.6354, p = 0.0025). Furthermore, there was a trend of a negative correlation between XDH and PD1 + immune cell infiltration (Fig. 5L). These findings suggest that the expression of XDH may trigger a cytotoxic immune response in HCC.

### The prognostic implication of the XDH-associated immune signature in HCC

**Fig. 6:**
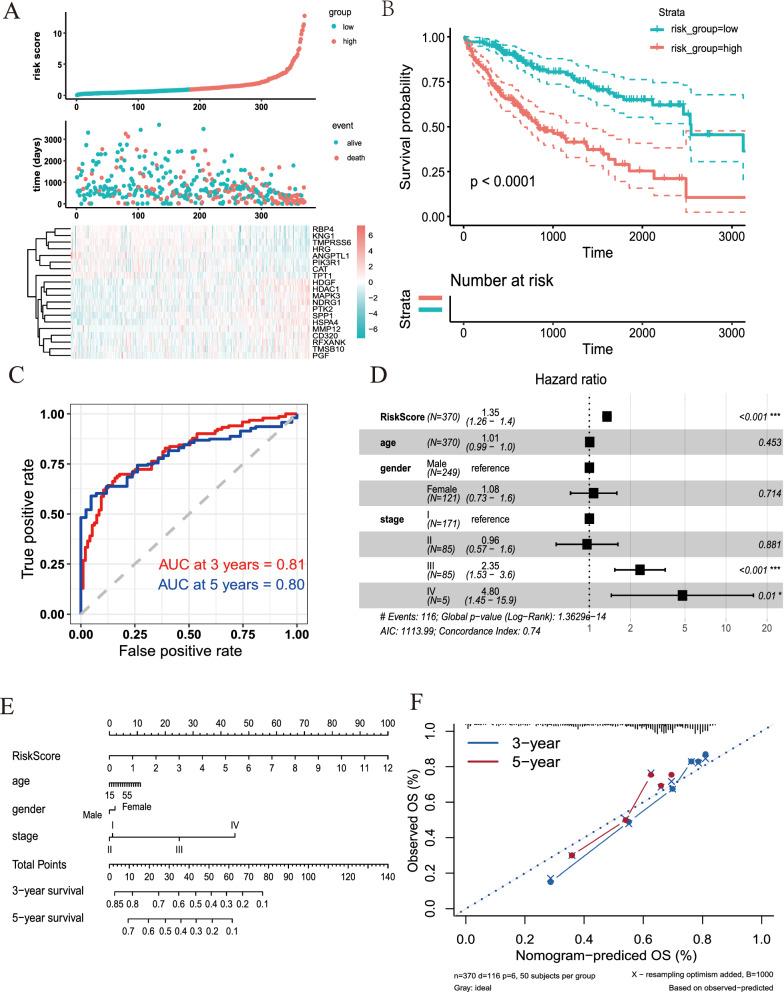
Prognostic value of the risk score in the TCGA-LIHC cohort. **A** Distribution of risk scores, survival statuses, and gene expression profiles for the LIHC cohort. **B** Kaplan–Meier curves for the LIHC patients stratified according to risk score. **C** Time-dependent ROC curves for 3- and 5-year survival. **D** Multivariate Cox regression analysis of the risk score of LIHC patients in terms of OS. **E** A nomogram for predicting the possible 3- and 5-year survival of individual LIHC patients. **F** The calibration curve of 3- and 5-year survival for the LIHC cohort. The 45° dashed line represents complete agreement between the nomogram-predicted values and real values

To further study the prognostic value of XDH, 218 immune-related genes were identified to be significantly associated with XDH expression by Pearson correlation analysis. The protein–protein network generated with the Search Tool for the Retrieval of Interacting Genes/Proteins (STRING) online server showed a tight correlation (Additional file 1: Figure S3). Then, a stepwise multivariable Cox regression analysis was used to identify the prognostic value of the XDH-associated immune signature in HCC. Consequently, an optimal prognostic signature of 20 genes related to XDH in HCC was revealed. The biological functions of these genes are presented in Additional file 1: Table S5. The distribution of risk scores, survival status, and signature gene expression profiles for HCC were visualized (Fig. 6A). Compared with patients with high risk scores, patients with low risk scores had a significantly longer survival time, as indicated by Kaplan–Meier survival curves (log-rank test, P < 0.001; Fig. 6B). In addition, the risk score showed a strong discriminative ability for 3-year and 5-year OS (Fig. 6C). After adjusting for sex, age, and stage, multivariable Cox regression analysis showed that the risk score was an independent predictor of prognosis in HCC (HR = 1.35, 95% CI = 1.26–1.4, p < 0.001; Fig. 6D). Finally, we built a prognostic nomogram for HCC patients by determining weighted coefficients for risk score, stage, age, and sex. The calibration curves showed that the nomogram-predicted probability (solid line) well matched the ideal reference line for 3- and 5-year survival (Fig. 6E, F). In addition, the prognostic nomogram displayed good discrimination with a C-index of 0.73. These data show that the XDH-associated immune signature has probable prognostic value in HCC.

## Discussion

The recent success of immune-based therapies has revolutionized the HCC treatment armamentarium [51–54]. However, there are very few useful biomarkers available for the identification of sensitivity and resistance to checkpoint inhibitors and their combinations in HCC [52]. In this study, we found that *XDH* mRNA levels correlated with the prognosis of several human cancers. In HCC, the downregulation of XDH was an independent survival predictor associated with worse prognosis. The XDH-related cell signaling pathway was associated with a cytotoxic immune response in HCC. Furthermore, XDH mRNA levels correlated with the numbers of tumor-infiltrated immune cells based on the levels of markers for different immune cell types in HCC. The prognostic implication of the XDH-associated immune signature in HCC supports that XDH is a potential immune biomarker for HCC.

In the present study, we first comprehensively analyzed the mRNA levels of *XDH* and their prognostic value in cancer tissues using multiple databases. We also found that XDH expression was significantly downregulated in certain types of cancer, while increased XDH expression was detected in some other cancers, such as HNSC, LUAD, and LUSC. Indeed, the XDH expression data were consistent in HCC tissues across different databases. Thus, the variability of XDH expression in different types of cancers may reflect differences in the underlying molecular and genetic mechanisms for cancer development and progression. Although the prognostic potential of XDH in several types of cancer has been reported, our work expands this knowledge of XDH in other cancers. Indeed, analysis of the GEPIA database revealed that low XDH expression was correlated with a worse prognosis in cancer types such as CHOL, lower grade glioma (LGG), LUAD, and thyroid carcinoma (THCA).

We hypothesized that XDH could play a significant role in regulating tumor immunology and therefore influence the outcomes of cancer patients. Analysis of the TIMER database revealed that XDH expression correlated with the infiltration status of immune cells in several cancer types, including HCC. In HCC, a positive correlation was found between XDH expression and CD8 + T-cell infiltration in the analysis of both the TIMER database and IHC staining of immune cell markers in HCC tumor tissues. Although there was a significant positive correlation between XDH expression and macrophage infiltration in the TIMER database, only a positive trend was found between XDH expression and CD68 + macrophage infiltration in this small group of HCC tissues. Indeed, Saidak et al. indicated that XDH expression is linked to an immune infiltrate in tumors [55]. XDH expression initiates several immune-related pathways, including the T cell receptor, PI3K-AKT, and MAPK signaling pathways. Similarly, both Wang et al. and Peng et al. found that immune-related pathways in HCC were mainly involved in the MAPK signaling pathway and PI3K-AKT signaling pathway [56, 57]. Moreover, the expression of the exhausted T cell markers PD-1 and CTLA-4, which are critical inhibitory immune checkpoint proteins, negatively correlated with XDH expression. Thus, decreased expression of XDH may facilitate tumor invasion in HCC and possibly in other similar cancers with low XDH expression. However, this hypothesis warrants further investigation.

The development of well-verified signatures for cancer prognosis evaluation represents a critical step for the implementation of stratification strategies and personalized immunotherapy for cancer [58–61]. In this study, we constructed a nomogram for personalized prognosis prediction in HCC with a C-index of 0.73. The risk scores derived from the XDH-associated immune gene signatures were significantly associated with survival. Notably, most of the immune genes integrated into the prognostic signatures participate in the regulation of the activity of immune cells, highlighting the significance of cytotoxic activity in HCC. Indeed, several prognosis-related risk models have been established according to the TCGA liver cancer cohort [62–67]. For instance, among 6 developed models, the 10-gene model proposed by Zhao et al. reached the highest C-index of 0.715 [62]. Similarly, Xu et al. established an 8-immune-gene prognostic signature with a C-index of 0.725 for HCC [68]. Recently, Chen et al. found nine immune-related gene pairs (IRGPs) that could be used to determine the outcomes of HCC patients with a C-index of 0.755 by integrating three public datasets of HCC 67. Moreover, some other predication models with high C-index values have been reported [56]. Nevertheless, none of those studies had risk models that reached area under the curve (AUC) values as high as 0.81 and 0.802 for 3-year and 5-year OS, respectively, as indicated by our model.

Our study has some limitations to be improved. First, this study is mainly based on data retrieved from public databases. Some in vitro or in vivo experiments are necessary to validate the immune evasion mechanism by which XDH contributes to the progression of HCC. Second, the sample size for some specific tumor types including HCC was too small to form a solid conclusion. Hence, further studies are necessary to verify the role of XDH in the regulation of immune-related pathways in HCC.

## Conclusion

In summary, our results suggest that XDH is a potential independent prognostic biomarker for HCC. The XDH-associated cell signaling pathway may affect immune cell infiltration into the tumor microenvironment. In HCC, decreased XDH expression correlates with a reduced cytotoxic immune response, and our prognostic XDH-associated immune signature provides a valuable tool for precision therapy. These data may indicate that XDH plays a role in HCC tumor immunology via an immune evasion mechanism.

## Supplementary Information

**Additional file 1:Figure S1.** Correlation of xanthine dehydrogenase (XDH) expression with prognosis in 25 diverse types of cancer. Overall survival (OS) and disease-free survival (DFS) curves comparing the high and low XDH expression groups in the BLCA (A-B), BRCA (C-D), CESC (E-F), CHOL (G-H), COAD (I-J), ESCA (K-L), GBM (M-N), HNSC (O-P), KIRC (Q-R), KIRP (S-T), LIHC (U-V), LUSC (W-X), MESO (Y-Z), OV (AA-AB), PAAD (AC-AD), PRAD (AE-AF), READ (AG-AH), SARC (AI-AJ), SKCM (AK-AL), STAD (AM-AN), TGCT (AO-AP), THCA (AQ-AR), THYM (AS-AT), UCEC (AU-AV), and UCS (AW-AX) cohorts. **Figure S2.** Quantification of western blotting results in Fig. 5F. **Figure S3.** Representative images of CD3 (A), CD4 (B), CD8 (C), CD20 (D), CD68 (E), PD1 (F) and XDH (G) staining. **Figure S4.** Protein-protein network of 128 XDH-associated immune genes in LIHC produced by the STRING online server. **Table S1.** List of the primary antibodies and dilutions used in the study. **Table S2.** Antibody sources and staining conditions. **Table S3.** XDH expression in cancer tissues versus normal tissues in the bladder cancer, breast cancer, colorectal cancer, head and neck cancer, leukemia, liver cancer, lung cancer, and lymphoma datasets in the Oncomine database. **Table S4.** Correlation of XDH expression with immune infiltration levels in diverse types of cancer in the Tumor IMmune Estimation Resource (TIMER) database. **Table S5.** Functions of the genes included in the prognostic signature.

**Additional file 2:** Immunostaining score.

**Additional file 3:** WB quantification.

## Data Availability

All data generated or analyzed during this study are included in this article and its Additional file 1, 2, 3.

## Abbreviations

XDH: Xanthine dehydrogenase
HCC: Hepatocellular carcinoma
TIMER: Tumor immune estimation resource
TCGA: The Cancer Genome Atlas
IHC: Immunohistochemistry
PVDF: Polyvinylidene difluoride
GEPIA: Gene expression profiling interactive analysis
GTEx: Genotype-tissue expression
DCs: Dendritic cells
OS: Overall survival
DFS: Disease-free survival
RFS: Recurrence-free survival
PFS: Progression-free survival
KEGG: Kyoto Encyclopedia of Genes and Genomes
GO: Gene ontology
TNM: Tumor-node-metastasis
DSS: Disease-specific survival
ROC: Receiver operating characteristic
HR: Hazard ratio
CI: Confidence interval
PD1: Programmed cell death protein 1
CTLA4: Cytotoxic T-lymphocyte-associated protein 4
TIM3: T cell immunoglobulin and mucin domain-containing protein 3
LAG-3: Lymphocyte-activation gene 3
STRING: Search Tool for the Retrieval of Interacting Genes/Proteins
AUC: Area under the curve
ACC: Adrenocortical carcinoma
BLCA: Bladder urothelial carcinoma
BRCA: Breast invasive carcinoma
CESC: Cervical squamous cell carcinoma
CHOL: Cholangiocarcinoma
COAD: Colon adenocarcinoma
DLBC: Diffuse large b-cell lymphoma
ESCA: Esophageal carcinoma
GBM: Glioblastoma multiforme
HNSC: Head and neck squamous cell carcinoma
KIRC: Kidney renal clear cell carcinoma
KIRP: Kidney renal papillary cell carcinoma
LIHC: Liver hepatocellular carcinoma
LUSC: Lung squamous cell carcinoma
LGG: Lower grade glioma
MESO: Mesothelioma
OV: Ovarian serous cystadenocarcinoma
PAAD: Pancreatic adenocarcinoma
PCPG: Pheochromocytoma and paraganglioma
PRAD: Prostate adenocarcinoma
READ: Rectum adenocarcinoma
SARC: Sarcoma
SKCM: Skin cutaneous melanoma
STAD: Stomach adenocarcinoma
TGCT: Testicular germ cell tumor
THCA: Thyroid carcinoma
THYM: Thymoma
UCEC: Uterine corpus endometrial carcinoma
USC: Uterine carcinosarcoma
UVM: Uveal melanoma
