# Supplementary material for: Xanthine dehydrogenase as a prognostic biomarker related to tumor immunology in hepatocellular carcinoma
Source: Cancer Cell Int. 2021 Sep 8;21:475. doi: 10.1186/s12935-021-02173-7 (PMC8425161; doi:10.1186/s12935-021-02173-7)
Supplement: Supplementary file 1 — Additional file 1:Figure S1. Correlation of xanthine dehydrogenase (XDH) expression with prognosis in 25 diverse types of cancer. Overall survival (OS) and disease-free survival (DFS) curves comparing the high and low XDH expression groups in the BLCA (A-B), BRCA (C-D), CESC (E-F), CHOL (G-H), COAD (I-J), ESCA (K-L), GBM (M-N), HNSC (O-P), KIRC (Q-R), KIRP (S-T), LIHC (U-V), LUSC (W-X), MESO (Y-Z), OV (AA-AB), PAAD (AC-AD), PRAD (AE-AF), READ (AG-AH), SARC (AI-AJ), SKCM (AK-AL), STAD (AM-AN), TGCT (AO-AP), THCA (AQ-AR), THYM (AS-AT), UCEC (AU-AV), and UCS (AW-AX) cohorts. Figure S2. Quantification of western blotting results in Fig. 5F. Figure S3. Representative images of CD3 (A), CD4 (B), CD8 (C), CD20 (D), CD68 (E), PD1 (F) and XDH (G) staining. Figure S4. Protein-protein network of 128 XDH-associated immune genes in LIHC produced by the STRING online server. Table S1. List of the primary antibodies and dilutions used in the study. Table S2. Antibody sources and staining conditions. Table S3. XDH expression in cancer tissues versus normal tissues in the bladder cancer, breast cancer, colorectal cancer, head and neck cancer, leukemia, liver cancer, lung cancer, and lymphoma datasets in the Oncomine database. Table S4. Correlation of XDH expression with immune infiltration levels in diverse types of cancer in the Tumor IMmune Estimation Resource (TIMER) database. Table S5. Functions of the genes included in the prognostic signature. [file 12935_2021_2173_MOESM1_ESM.pdf]

Supplementary Figure1

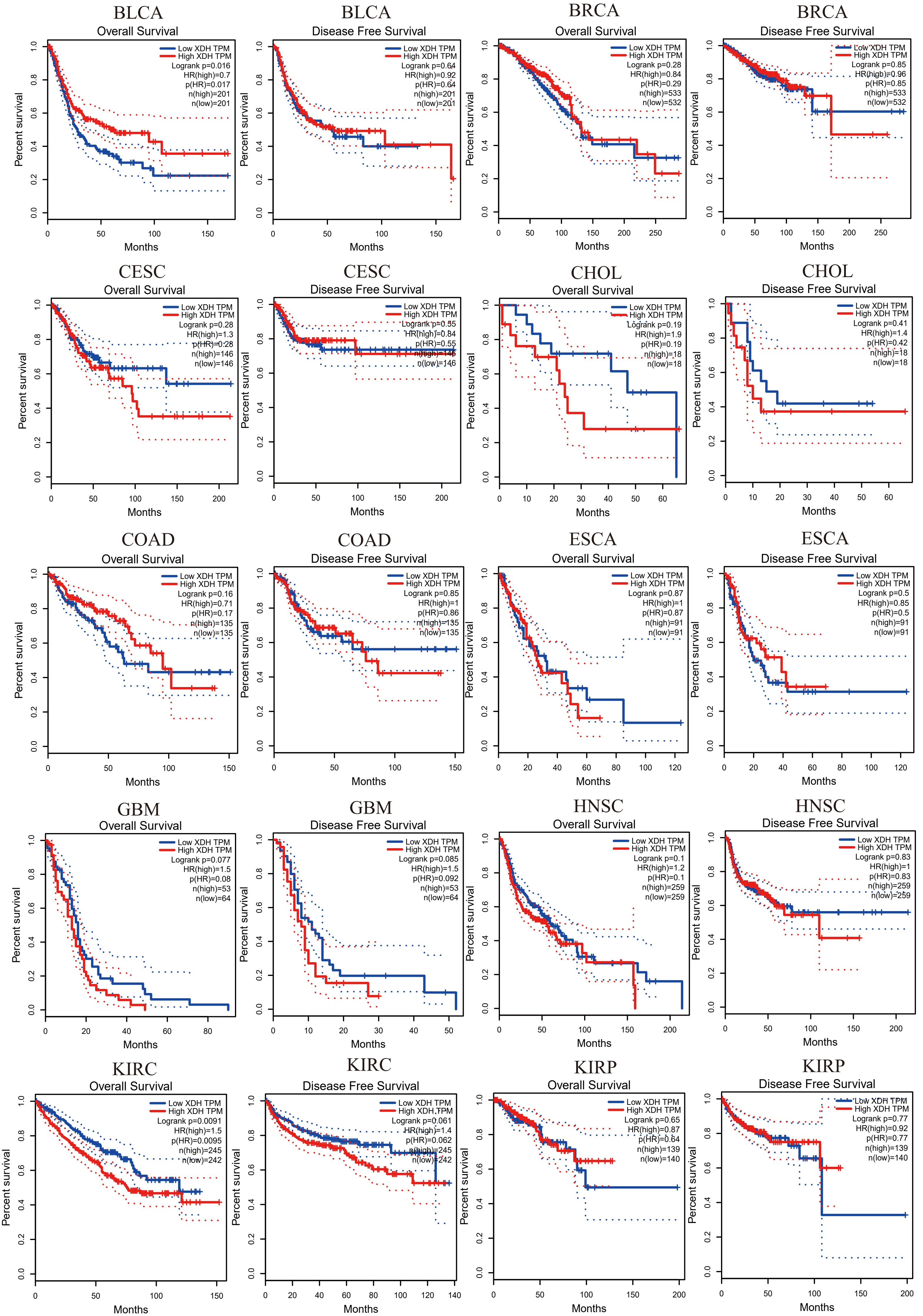

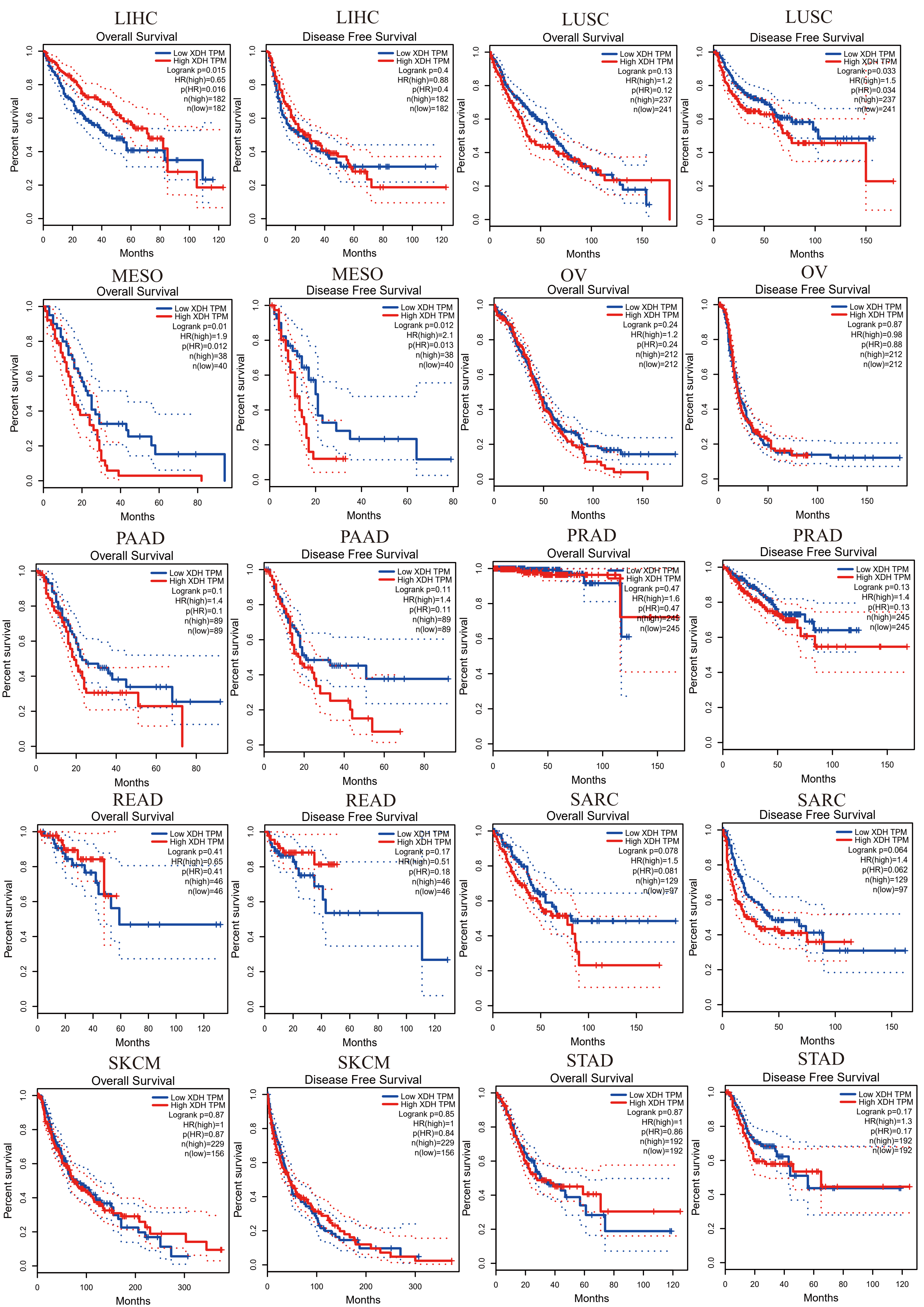
LIHC
Overall Survival
Low XDH TPM
High XDH TPM
Logrank p=0.015
HR(high)=0.65
p(HR)=0.016
n(high)=182
n(low)=182
LIHC
Disease Free Survival
Low XDH TPM
High XDH TPM
Logrank p=0.4
HR(high)=0.88
p(HR)=0.4
n(high)=182
n(low)=182
LUSC
Overall Survival
Low XDH TPM
High XDH TPM
Logrank p=0.13
HR(high)=1.2
p(HR)=0.12
n(high)=237
n(low)=241
LUSC
Disease Free Survival
Low XDH TPM
High XDH TPM
Logrank p=0.033
HR(high)=1.5
p(HR)=0.034
n(high)=237
n(low)=241
MESO
Overall Survival
Low XDH TPM
High XDH TPM
Logrank p=0.01
HR(high)=1.9
p(HR)=0.012
n(high)=38
n(low)=40
MESO
Disease Free Survival
Low XDH TPM
High XDH TPM
Logrank p=0.012
HR(high)=2.1
p(HR)=0.013
n(high)=38
n(low)=40
OV
Overall Survival
Low XDH TPM
High XDH TPM
Logrank p=0.24
HR(high)=1.2
p(HR)=0.24
n(high)=212
n(low)=212
OV
Disease Free Survival
Low XDH TPM
High XDH TPM
Logrank p=0.87
HR(high)=0.98
p(HR)=0.88
n(high)=212
n(low)=212
PAAD
Overall Survival
Low XDH TPM
High XDH TPM
Logrank p=0.1
HR(high)=1.4
p(HR)=0.1
n(high)=89
n(low)=89
PAAD
Disease Free Survival
Low XDH TPM
High XDH TPM
Logrank p=0.11
HR(high)=1.4
p(HR)=0.11
n(high)=89
n(low)=89
PRAD
Overall Survival
Low XDH TPM
High XDH TPM
Logrank p=0.47
HR(high)=1.6
p(HR)=0.47
n(high)=245
n(low)=245
PRAD
Disease Free Survival
Low XDH TPM
High XDH TPM
Logrank p=0.13
HR(high)=1.4
p(HR)=0.13
n(high)=245
n(low)=245
READ
Overall Survival
Low XDH TPM
High XDH TPM
Logrank p=0.41
HR(high)=0.65
p(HR)=0.41
n(high)=46
n(low)=46
READ
Disease Free Survival
Low XDH TPM
High XDH TPM
Logrank p=0.17
HR(high)=0.51
p(HR)=0.18
n(high)=46
n(low)=46
SARC
Overall Survival
Low XDH TPM
High XDH TPM
Logrank p=0.078
HR(high)=1.5
p(HR)=0.081
n(high)=129
n(low)=97
SARC
Disease Free Survival
Low XDH TPM
High XDH TPM
Logrank p=0.064
HR(high)=1.4
p(HR)=0.062
n(high)=129
n(low)=97
SKCM
Overall Survival
Low XDH TPM
High XDH TPM
Logrank p=0.87
HR(high)=1
p(HR)=0.87
n(high)=229
n(low)=156
SKCM
Disease Free Survival
Low XDH TPM
High XDH TPM
Logrank p=0.85
HR(high)=1
p(HR)=0.84
n(high)=229
n(low)=156
STAD
Overall Survival
Low XDH TPM
High XDH TPM
Logrank p=0.87
HR(high)=1
p(HR)=0.86
n(high)=192
n(low)=192
STAD
Disease Free Survival
Low XDH TPM
High XDH TPM
Logrank p=0.17
HR(high)=1.3
p(HR)=0.17
n(high)=192
n(low)=192
Percent survival
Months

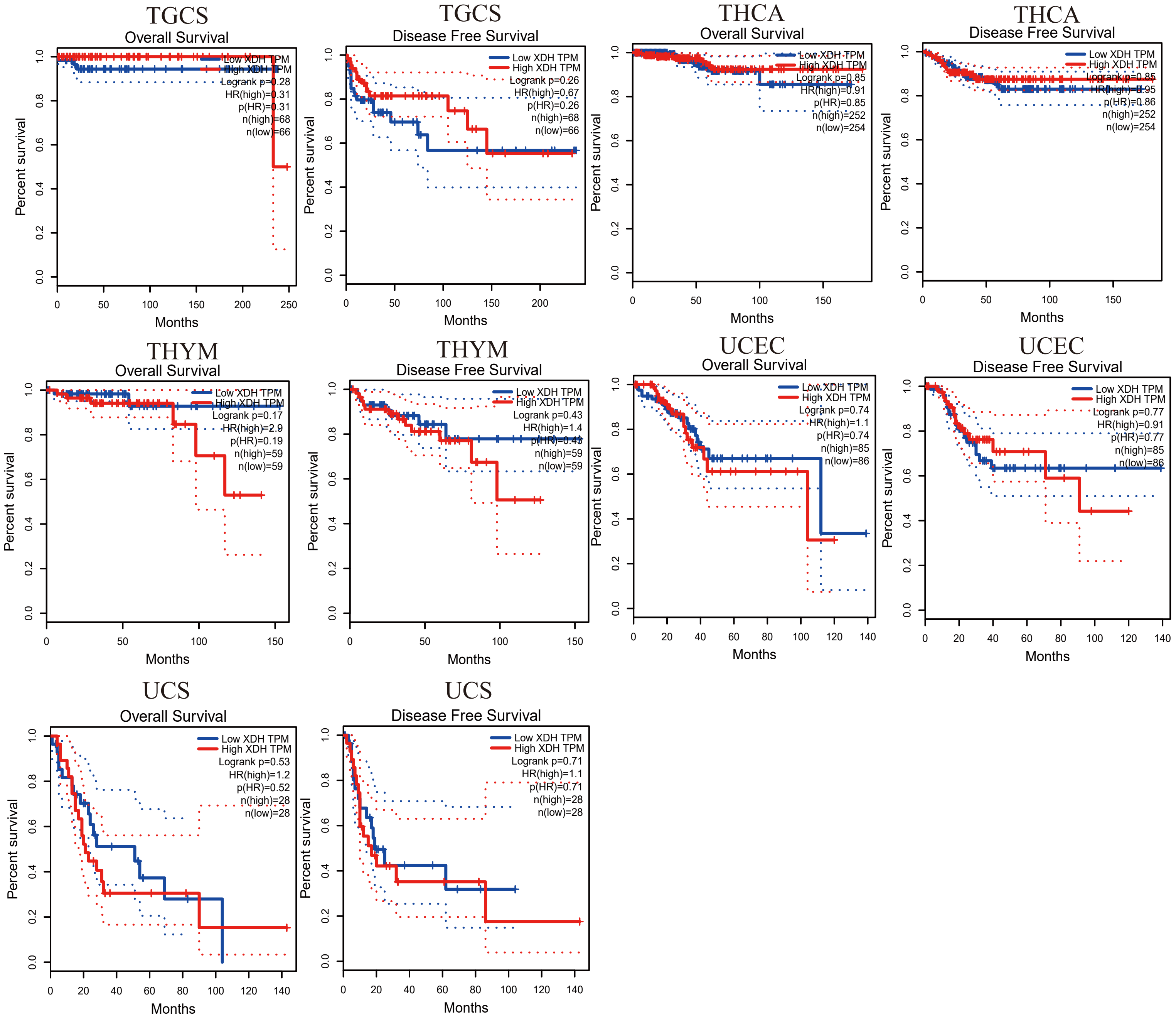
TGCS
Overall Survival
Low XDH TPM
High XDH TPM
Logrank p=0.28
HR(high)=0.31
p(HR)=0.31
n(high)=68
n(low)=66
Percent survival
Months
TGCS
Disease Free Survival
Low XDH TPM
High XDH TPM
Logrank p=0.26
HR(high)=0.67
p(HR)=0.26
n(high)=68
n(low)=66
Percent survival
Months
THCA
Overall Survival
Low XDH TPM
High XDH TPM
Logrank p=0.85
HR(high)=0.91
p(HR)=0.85
n(high)=252
n(low)=254
Percent survival
Months
THCA
Disease Free Survival
Low XDH TPM
High XDH TPM
Logrank p=0.85
HR(high)=0.95
p(HR)=0.86
n(high)=252
n(low)=254
Percent survival
Months
THYM
Overall Survival
Low XDH TPM
High XDH TPM
Logrank p=0.17
HR(high)=2.9
p(HR)=0.19
n(high)=59
n(low)=59
Percent survival
Months
THYM
Disease Free Survival
Low XDH TPM
High XDH TPM
Logrank p=0.43
HR(high)=1.4
p(HR)=0.43
n(high)=59
n(low)=59
Percent survival
Months
UCEC
Overall Survival
Low XDH TPM
High XDH TPM
Logrank p=0.74
HR(high)=1.1
p(HR)=0.74
n(high)=85
n(low)=86
Percent survival
Months
UCEC
Disease Free Survival
Low XDH TPM
High XDH TPM
Logrank p=0.77
HR(high)=0.91
p(HR)=0.77
n(high)=85
n(low)=86
Percent survival
Months
UCS
Overall Survival
Low XDH TPM
High XDH TPM
Logrank p=0.53
HR(high)=1.2
p(HR)=0.52
n(high)=28
n(low)=28
Percent survival
Months
UCS
Disease Free Survival
Low XDH TPM
High XDH TPM
Logrank p=0.71
HR(high)=1.1
p(HR)=0.71
n(high)=28
n(low)=28
Percent survival
Months

# Supplementary Figure 2

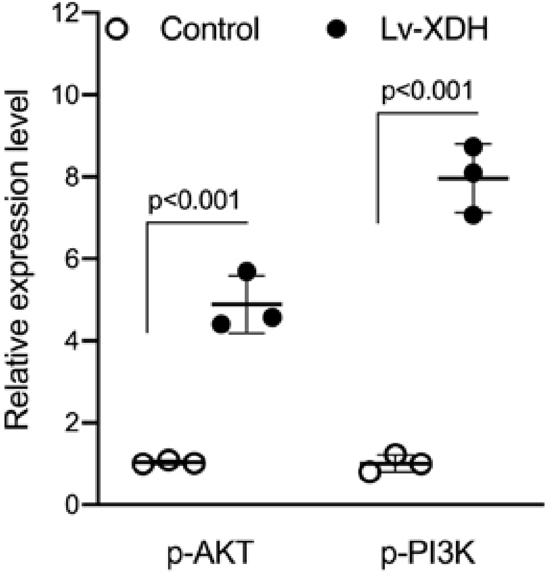

# Supplementary Figure 3

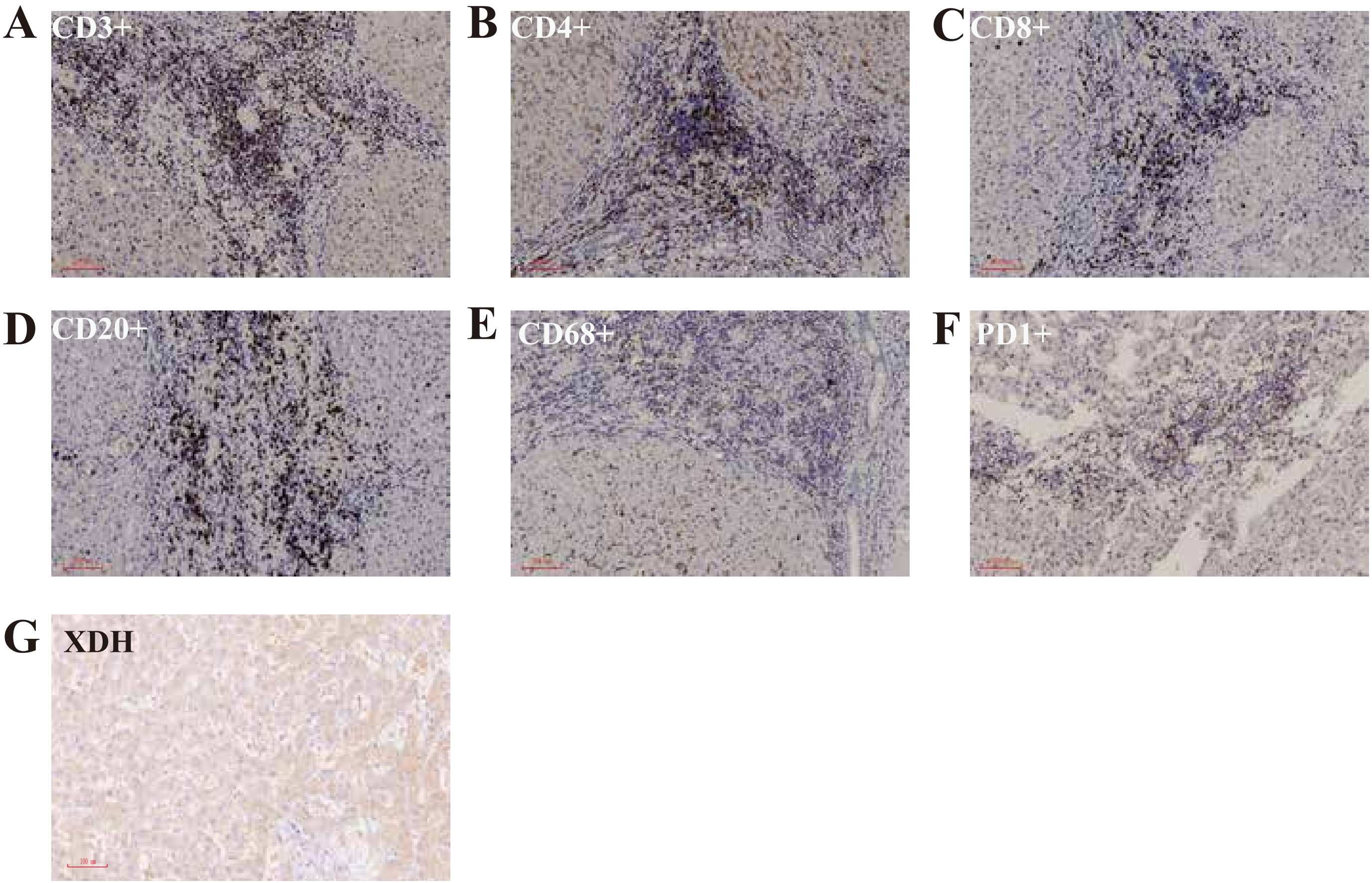

# Supplementary Figure 4

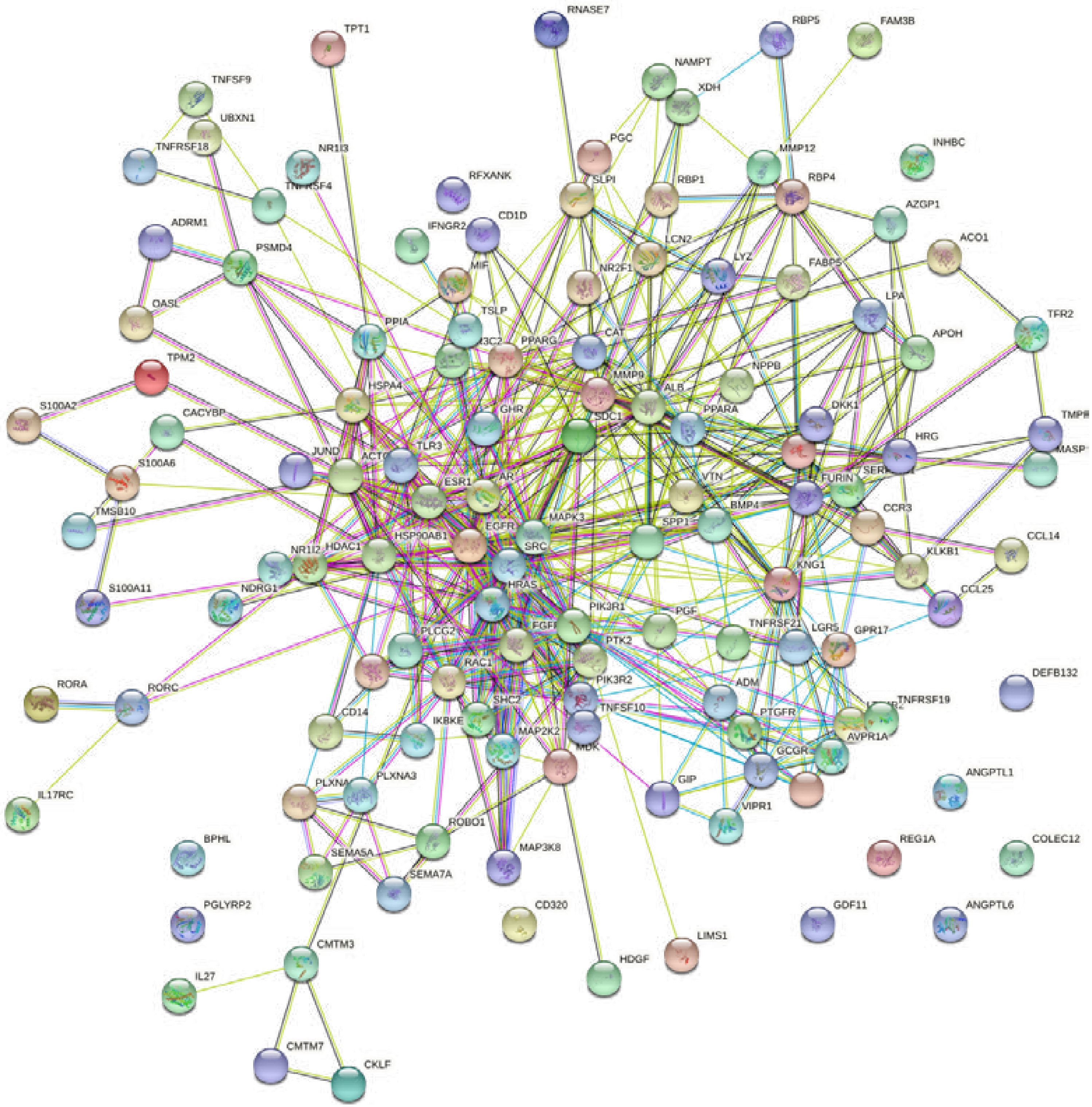

Supplementary Table 1

| Antibody | Source | Catalogue | Dilution |
|---|---|---|---|
| α-Tubulin | GeneTex Inc. | #GTX102078 | 1:1000 |
| AKT | Cell Signaling Technology, Inc. | #9272 | 1:1000 |
| p-AKT | Cell Signaling Technology, Inc. | #4060 | 1:1000 |
| PI3K | Cell Signaling Technology, Inc. | #4249 | 1:1000 |
| p-PI3K | Cell Signaling Technology, Inc. | #4228 | 1:1000 |
| XDH | Santa Cruz Biotechnology | #sc-398548 | 1:1000 |

Supplementary Table 2

| Markers | Antibody source | Species | Dilution |
|---|---|---|---|
| CD3 | MXB Biotechnologies, MAB0740 | Mouse monoclonal | 1:200 |
| CD4 | MXB Biotechnologies, MAB-0251 | Mouse monoclonal | 1:200 |
| CD8 | MXB Biotechnologies, MAB-0021 | Mouse monoclonal | 1:200 |
| CD20 | MXB Biotechnologies, MAB-0669 | Mouse monoclonal | 1:200 |
| CD68 | MXB Biotechnologies, Kit-0026 | Mouse monoclonal | 1:200 |
| PD-1 | MXB Biotechnologies, MAB-0734 | Mouse monoclonal | 1:50 |

# Supplementary Table 3

| Cancer | Cancer Type | P value | Fold change | Rank (%) | Sample | Reference |
|---|---|---|---|---|---|---|
| Bladder | Superficial Bladder Cancer | 9.05E-4 | -2.181 | 5% | 54 | 15161696 |
| Breast | Male Breast Carcinoma | 1.51E-4 | -2.082 | 6% | 593 | TCGA |
| Colorectal | Rectosigmoid Adenocarcinoma | 1.96E-8 | -10.885 | 3% | 237 | TCGA |
| | Rectal Adenocarcinoma | 1.60E-14 | -4.390 | 8% | 237 | TCGA |
| | Colorectal Carcinoma | 1.22E-12 | -3.043 | 3% | 82 | 20143136 |
| | Colorectal Carcinoma | 9.87E-10 | -2.613 | 4% | 105 | 20957034 |
| | Colorectal Adenocarcinoma | 1.07E-7 | -2.158 | 5% | 105 | 20957034 |
| | Colon Adenoma | 1.11E-6 | -2.184 | 6% | 64 | 18171984 |
| Head and Neck Cancer | Oral Cavity Squamous Cell Carcinoma | 6.20E-7 | 2.163 | 9% | 79 | 21853135 |
| Leukemia | T-Cell Acute Lymphoblas c Leukemia | 1.14E-5 | -2.597 | 10% | 127 | 10663620 |
| Liver | Hepatocellular Carcinoma | 2.87E-9 | -5.058 | 1% | 75 | 17393520 |
| | Hepatocellular Carcinoma | 4.56E-9 | -3.046 | 2% | 43 | 21159642 |
| | Hepatocellular Carcinoma | 5.40E-50 | -2.989 | 3% | 445 | 21159642 |
| | Hepatocellular Carcinoma | 5.20E-12 | -2.551 | 6% | 197 | 12058060 |
| Lung | Lung Adenocarcinoma | 7.12E-17 | 2.229 | 1% | 246 | 22327624 |
| Lymphoma | Anaplas c Large Cell Lymphoma | 2.10E-5 | -2.379 | 9% | 60 | 18492688 |

# Supplementary Table 4

| Cancer | Purity | | B cell | | CD8+ T cell | | CD4+ T cell | | Macrophage | | Neutrophil | | dendritic cell | |
|---|---|---|---|---|---|---|---|---|---|---|---|---|---|---|
| | rho | p | rho | p | rho | p | rho | p | rho | p | rho | p | rho | p |
| ACC (n=79) | -0.011 | 0.926 | -0.053 | 0.657 | -0.065 | 0.584 | 0.009 | 0.939 | 0.134 | 0.257 | 0.188 | 0.111 | -0.246 | **0.036** |
| BLCA (n=408) | -0.100 | 0.055 | 0.046 | 0.381 | 0.200 | **0.000** | -0.005 | 0.928 | -0.233 | **0.000** | 0.337 | **0.000** | 0.292 | **0.000** |
| BRCA (n=1100) | -0.194 | **0.000** | -0.159 | **0.000** | 0.081 | **0.010** | 0.017 | 0.602 | 0.032 | 0.315 | 0.204 | **0.000** | 0.094 | **0.003** |
| BRCA-Basal (n=191) | -0.120 | 0.114 | -0.197 | **0.009** | 0.137 | 0.071 | -0.168 | **0.027** | 0.160 | **0.035** | 0.117 | 0.124 | -0.093 | 0.224 |
| BRCA-Her2 (n=82) | -0.020 | 0.863 | -0.042 | 0.728 | -0.049 | 0.680 | 0.035 | 0.771 | 0.062 | 0.604 | 0.266 | **0.024** | 0.273 | **0.020** |
| BRCA-LumA (n=568) | -0.173 | **0.000** | -0.130 | **0.003** | 0.093 | **0.034** | 0.012 | 0.782 | 0.039 | 0.371 | 0.157 | **0.000** | 0.069 | 0.117 |
| BRCA-LumB (n=219) | -0.069 | 0.344 | -0.119 | 0.101 | 0.184 | **0.011** | 0.027 | 0.706 | 0.094 | 0.195 | 0.109 | 0.134 | 0.020 | 0.783 |
| CESC (n=306) | -0.208 | **0.000** | 0.043 | 0.478 | 0.184 | **0.002** | 0.008 | 0.898 | -0.018 | 0.766 | 0.258 | **0.000** | 0.279 | **0.000** |
| CHOL (n=36) | 0.018 | 0.917 | -0.039 | 0.822 | 0.126 | 0.471 | -0.052 | 0.765 | 0.268 | 0.119 | 0.240 | 0.165 | -0.079 | 0.652 |
| COAD (n=458) | -0.153 | **0.002** | 0.085 | 0.161 | 0.186 | **0.002** | 0.003 | 0.961 | -0.098 | 0.107 | 0.165 | **0.006** | 0.118 | 0.051 |
| DLBC (n=48) | -0.201 | 0.202 | -0.112 | 0.487 | 0.201 | 0.208 | -0.088 | 0.583 | 0.074 | 0.646 | 0.103 | 0.522 | -0.042 | 0.793 |
| ESCA (n=185) | -0.138 | 0.065 | -0.074 | 0.321 | 0.028 | 0.710 | -0.019 | 0.797 | -0.239 | **0.001** | 0.144 | 0.054 | 0.047 | 0.531 |
| GBM (n=153) | -0.086 | 0.317 | -0.011 | 0.895 | 0.125 | 0.146 | 0.030 | 0.731 | 0.096 | 0.267 | 0.158 | 0.065 | 0.294 | **0.000** |
| HNSC (n=522) | -0.247 | **0.000** | 0.103 | **0.022** | 0.285 | **0.000** | -0.023 | 0.606 | -0.008 | 0.861 | 0.168 | **0.000** | 0.295 | **0.000** |
| HNSC-HPV- (n=422) | -0.248 | **0.000** | 0.088 | 0.077 | 0.267 | **0.000** | 0.019 | 0.711 | -0.027 | 0.594 | 0.206 | **0.000** | 0.282 | **0.000** |
| HNSC-HPV+ (n=98) | -0.129 | 0.227 | 0.149 | 0.163 | 0.254 | **0.016** | -0.089 | 0.408 | 0.016 | 0.879 | 0.077 | 0.473 | 0.312 | **0.003** |
| KICH (n=66) | -0.245 | **0.048** | 0.075 | 0.551 | 0.011 | 0.929 | 0.248 | **0.046** | 0.085 | 0.502 | 0.184 | 0.141 | 0.299 | **0.015** |
| KIRC (n=533) | -0.168 | **0.000** | 0.016 | 0.733 | -0.004 | 0.924 | -0.028 | 0.548 | 0.005 | 0.919 | 0.067 | 0.152 | 0.156 | **0.001** |
| KIRP (n=290) | -0.166 | **0.007** | 0.060 | 0.333 | 0.218 | **0.000** | 0.121 | 0.052 | -0.006 | 0.921 | 0.298 | **0.000** | 0.234 | **0.000** |
| LGG (n=516) | 0.010 | 0.829 | -0.053 | 0.250 | -0.084 | **0.066** | 0.141 | **0.002** | 0.046 | 0.320 | 0.145 | **0.001** | 0.151 | **0.001** |
| LIHC (n=371) | 0.119 | **0.026** | -0.178 | **0.001** | 0.157 | **0.003** | -0.104 | 0.053 | 0.009 | 0.871 | -0.088 | 0.101 | -0.179 | **0.001** |
| LUAD (n=515) | -0.162 | **0.000** | -0.069 | 0.126 | 0.018 | 0.693 | 0.061 | 0.174 | 0.051 | 0.254 | 0.230 | **0.000** | 0.332 | **0.000** |
| LUSC (n=501) | -0.263 | **0.000** | -0.236 | **0.000** | 0.025 | 0.579 | -0.008 | 0.856 | -0.062 | 0.177 | 0.152 | **0.001** | 0.153 | **0.001** |
| MESO (n=87) | -0.117 | 0.283 | 0.401 | **0.000** | 0.076 | 0.491 | 0.109 | 0.320 | -0.001 | 0.994 | 0.016 | 0.882 | 0.290 | **0.007** |
| OV (n=303) | -0.180 | **0.004** | 0.086 | 0.176 | 0.164 | **0.010** | -0.091 | 0.154 | 0.209 | **0.001** | 0.270 | **0.000** | 0.118 | 0.063 |
| PAAD (n=179) | -0.116 | 0.129 | 0.255 | **0.001** | -0.031 | 0.690 | 0.018 | 0.815 | -0.046 | 0.551 | 0.054 | 0.483 | 0.073 | 0.342 |
| PCPG (n=181) | -0.016 | 0.834 | 0.077 | 0.325 | 0.150 | 0.053 | -0.164 | **0.034** | 0.150 | 0.054 | 0.202 | **0.009** | 0.139 | 0.073 |
| PRAD (n=498) | -0.106 | **0.030** | 0.112 | **0.023** | 0.221 | **0.000** | 0.000 | 0.997 | 0.127 | **0.009** | 0.244 | **0.000** | 0.110 | **0.025** |
| READ (n=166) | -0.113 | 0.184 | 0.188 | 0.076 | 0.171 | 0.106 | -0.073 | 0.493 | -0.049 | 0.647 | 0.081 | 0.445 | 0.099 | 0.354 |
| SARC (n=260) | -0.202 | **0.002** | 0.029 | 0.652 | 0.299 | **0.000** | -0.298 | **0.000** | -0.084 | 0.193 | -0.047 | 0.468 | 0.206 | **0.001** |
| SKCM (n=471) | -0.053 | 0.258 | -0.076 | 0.104 | -0.024 | 0.607 | -0.228 | **0.000** | 0.030 | 0.522 | 0.011 | 0.810 | -0.059 | 0.206 |
| SKCM-Metastasis (n=368) | -0.005 | 0.929 | -0.030 | 0.574 | 0.039 | 0.460 | -0.204 | **0.000** | 0.087 | 0.101 | 0.063 | 0.235 | 0.026 | 0.631 |
| SKCM-Primary (n=103) | -0.243 | **0.013** | -0.156 | 0.118 | -0.127 | 0.204 | -0.057 | 0.568 | 0.015 | 0.882 | 0.261 | **0.008** | -0.157 | 0.115 |
| STAD (n=415) | -0.107 | **0.037** | 0.013 | 0.795 | -0.151 | **0.003** | -0.012 | 0.809 | -0.156 | **0.002** | 0.055 | 0.286 | -0.071 | 0.166 |
| TGCT (n=150) | 0.170 | **0.039** | 0.005 | 0.949 | -0.093 | 0.262 | 0.177 | **0.032** | 0.032 | 0.700 | 0.045 | 0.587 | -0.109 | 0.190 |
| THCA (n=509) | -0.056 | 0.217 | -0.175 | **0.000** | 0.009 | 0.849 | 0.055 | 0.229 | -0.189 | **0.000** | 0.520 | **0.000** | 0.627 | **0.000** |
| THYM (n=120) | 0.127 | 0.174 | -0.229 | **0.014** | -0.384 | 0.000 | -0.407 | **0.000** | 0.146 | 0.119 | -0.278 | **0.003** | -0.371 | **0.000** |
| UCEC (n=545) | -0.063 | 0.283 | -0.273 | **0.010** | -0.071 | 0.512 | -0.005 | 0.966 | -0.379 | **0.000** | 0.069 | 0.525 | 0.309 | **0.003** |
| UCS (n=57) | -0.203 | 0.140 | 0.214 | 0.124 | -0.195 | 0.162 | 0.247 | 0.074 | 0.349 | **0.011** | 0.395 | **0.003** | 0.167 | 0.232 |
| UVM (n=80) | 0.022 | 0.848 | -0.123 | 0.287 | 0.137 | 0.234 | -0.146 | 0.204 | 0.107 | 0.355 | 0.224 | 0.051 | -0.206 | 0.072 |

Supplementary Table 5

| Symbol | Name | Function |
|---|---|---|
| ANGPTL1 | angiopoietin-like 1 | Cytokine Receptors |
| CAT | catalase | Cytokines |
| CD320 | CD320 molecule | Cytokines |
| HDAC1 | histone deacetylase 1 | Adaptive immunity |
| HDGF | hepatoma-derived growth factor | Cytokines |
| HRG | histidine-rich glycoprotein | Adaptive immunity |
| HSPA4 | heat shock 70kDa protein 4 | Antigen Processing and Presentation |
| KNG1 | kininogen 1 | Adaptive immunity |
| MAPK3 | mitogen-activated protein kinase 3 | Natural Killer Cell Cytotoxicity |
| MMP12 | matrix metallopeptidase 12 | Adaptive immunity |
| NDRG1 | N-myc downstream regulated 1 | Adaptive immunity |
| PGF | placental growth factor | Cytokines |
| PIK3R1 | phosphoinositide-3-kinase, regulatory subunit 1 | TCR signaling Pathway; Natural Killer Cell Cytotoxicity |
| PTK2 | PTK2 protein tyrosine kinase 2 | Adaptive immunity |
| RBP4 | retinol binding protein 4, plasma | Adaptive immunity |
| RFXANK | regulatory factor X-associated ankyrin-containing protein | Antigen Processing and Presentation |
| SPP1 | secreted phosphoprotein 1 | Cytokines |
| TMPRSS6 | transmembrane protease, serine 6 | Adaptive immunity |
| TMSB10 | thymosin beta 10 | Adaptive immunity |
| TPT1 | tumor protein, translationally-controlled 1 | Adaptive immunity |
